# Peripheral nerve complications from medications utilised for weight loss: a systematic review

**DOI:** 10.1007/s00415-026-14048-w

**Published:** 2026-08-03

**Authors:** Cullen O’Gorman, Matilda O’Gorman, Nicola Warren

**Affiliations:** 1 Department of Neurology, Mater Hospital, Raymond Terrace, South Brisbane, Brisbane, QLD Australia; 2 University of Queensland, Brisbane, QLD Australia; 3 Queensland Centre for Mental Health Research, Wacol, Australia; 4 St. Margaret’s Anglican Girls School, Ascot, Queensland, Australia

**Keywords:** Neuropathy, Diabetes, Entrapment, Weight loss, Complication, GLP

## Abstract

**Background:**

There is a global epidemic of obesity and in this context, the development of novel agents for weight loss has been timely. There have been emerging reports of peripheral nerve complications and this study aimed to systematically review all reported cases associated with medications used for weight loss.

**Methods:**

A systematic search of PubMed, Embase and CINAHL databases from inception to 30 June 2026 was performed to identify studies that report new-onset or worsening peripheral nerve complications. Independent screening was conducted by two authors. Data including demographics, medications, weight-loss parameters, neurological presentation and investigations. Descriptive statistics were used.

**Results:**

There were 19 studies, comprising 31 cases and one cohort (103 individuals) identified. All bar one were published from 2024. Of 31 individual cases median weight loss achieved was 20.4 kg (IQR 15.2) or 21.8% (IQR 16.4) of starting body weight. Median rate of weight loss was 3.7 kg/month (IQR 1.7), with symptom onset at 150 days (IQR 135). Semaglutide or tirzepatide were implicated in the majority (83%), and 67% had pre-diabetes/diabetes. Neurological presentations included fibular neuropathy (*n* = 10), lumbosacral radiculoplexus neuropathy (*n* = 9) and distal symmetric polyneuropathy (*n* = 7). Electrodiagnostic and imaging data were supportive of diagnosis.

**Conclusions:**

There is a recent increase in peripheral nerve disorders associated with medical treatment of diabetes and obesity. To reduce the risk, it is recommended to ensure adequate nutritional status and avoid ultrarapid HbA1c and weight loss. Prospective studies are needed to determine incidence and confirm safe rates of weight loss and glycemic correction.

**Supplementary Information:**

The online version contains supplementary material available at 10.1007/s00415-026-14048-w.

## Highlights

- There is increasing use of novel hypoglycemic agents for diabetes control and weight loss.
- Rising number of reports of nerve complications associated with these novel agents
- Weight loss and rapid normalisation of hyperglycemia are more likely to cause nerve injury than direct toxicity from weight loss therapies

**Supplementary Information:**

The online version contains supplementary material available at 10.1007/s00415-026-14048-w.

## Introduction

Almost half the adult population worldwide is estimated to be overweight or obese, and forecasting projects a 30% increase over the next 30 years [1]. Obesity is considered a threat to global health progress [2] and is associated with substantial individual, community and population level costs [3]. However, achieving a sustained loss sufficient to improve metabolic outcomes is challenging. The majority of lifestyle and dietary interventions are associated with relatively poor adherence and have limited effect [4–6]. Bariatric surgery can provide sustained weight loss for selected populations, but surgical morbidity, mortality and the need for reoperation is not insignificant [7]. Pharmacotherapies for weight loss have been available for decades; however, the recent development of novel glucagon-like peptide-1 receptor agonists (GLP-1RA) has changed the landscape for obesity treatment [8].

GLP-1RAs demonstrate sustained reductions in weight, body mass index (BMI) and waist circumference, as well as improving obesity comorbidities [9, 10]. Studies have demonstrated between 5 and 19% reduction of body weight for participants over 52 weeks associated with GLP-1RA usage, greater in long-acting therapies and with the more recently available dual glucose-dependent insulinotropic polypeptide (GIP)/GLP-1RAs [11, 12]. Unsurprisingly, there has been considerable uptake of these therapies, with a 2025 United States survey reporting around 12% of adults having tried a GLP-1RA and an additional 14% expressing interest in doing so [13].

Peripheral nerve complications associated with significant weight loss were described by Paget in 1876, who reported on fibular neuropathy occurring in individuals with weight loss secondary to chronic infection [14]. Weight loss related neuropathies have also occurred in the context of intentional dieting, anorexia nervosa, starvation, cancer, thyrotoxicosis and weight loss surgery [15–22]. Postulated risk factors include rate, duration, and total amount of weight loss, as well as nutritional deficiency and comorbid medical concerns [15, 23, 24], with one study highlighting those losing more than 5 kg over a period of one month being at particular risk [23]. The spectrum of nerve disorders described includes mononeuropathy, distal sensory polyneuropathy and plexopathy. Although improvement with supportive care is seen in many, the majority of cases report long-term residual deficits [19].

Whilst a range of GLP-1RA adverse effects are well appreciated, literature on associated peripheral nerve complications is only recently emerging [26–28]. Yet, with both weight reduction efficacy and global use increasing, review to highlight presentation, risks, treatments and outcomes of these complications is vital. This study aims to systematically review all cases of peripheral nerve complications associated with medication that can be utilized for weight loss.

## Methods

The study was preregistered with PROSPERO (CRD420261354365) [29] and the Preferred Reporting Items for Systematic Reviews and Meta-Analyses (PRISMA) statement recommendations were followed [30].

### Search strategy

Databases PubMed, Embase and CINAHL were searched from inception to 30th June 2026 using terms to capture any peripheral nerve complication associated with weight loss medications including GLP-1RAs, dual GIP/GLP-1RA, sodium glucose transport protein-2 (SGLT2) inhibitors, phentermine, bupropion, topiramate and orlistat (see supplementary materials for full search terms). Search and screening at title, abstract and then full text was performed independently by two authors and references of selected articles were manually searched for additional studies. Any divergence was resolved by discussion.

Articles or abstracts were included if any cases, case series or cohorts described new onset or new deterioration of peripheral nerve complications consequent to use of any medication that can be used for weight loss. Weight loss was not required to be the primary indication for medication prescription. For the purposes of this review, any onset of peripheral nerve symptoms within 12 months of starting medication or weight loss occurring was considered potentially causative and included for further analysis. Given the emerging evidence in this field, case reports and conference abstracts were included if not superseded by later publication of findings. All languages were included. Studies reporting neurological complications in the central nervous system, or in association with surgical treatments or dietary modification alone (without use of medication), or weight loss consequent to systemic illness were excluded. Weight loss-associated rhabdomyolysis or myopathy were excluded.

### Data extraction and synthesis

Data extraction was completed independently by two authors including: research design, study location, demographics, diagnosis, data to support exclusion of other causes of neurological complication, medication details (dose, duration), degree and time frame of weight loss, available paraclinical data (results of screening blood tests, supportive imaging and electrophysiologic testing). Non-documentation of symptoms and diagnoses were considered absent, whereas non-documentation of investigations was considered as missing information and recorded as such. Descriptive statistics were used where possible to summarize information, using median and interquartile range for non–normally distributed data. Weight was converted to metric units where necessary. Rate of weight loss was calculated. Onset and progression of peripheral nerve complication was categorized as acute (hours to days), subacute (weeks), chronic (months) or insidious (more than 1 year). Quality assessment of all studies was performed using modified Johanna Briggs Institute (JBI) critical appraisal tools for case reports and cohort studies [31].

## Results

**Fig. 1:**
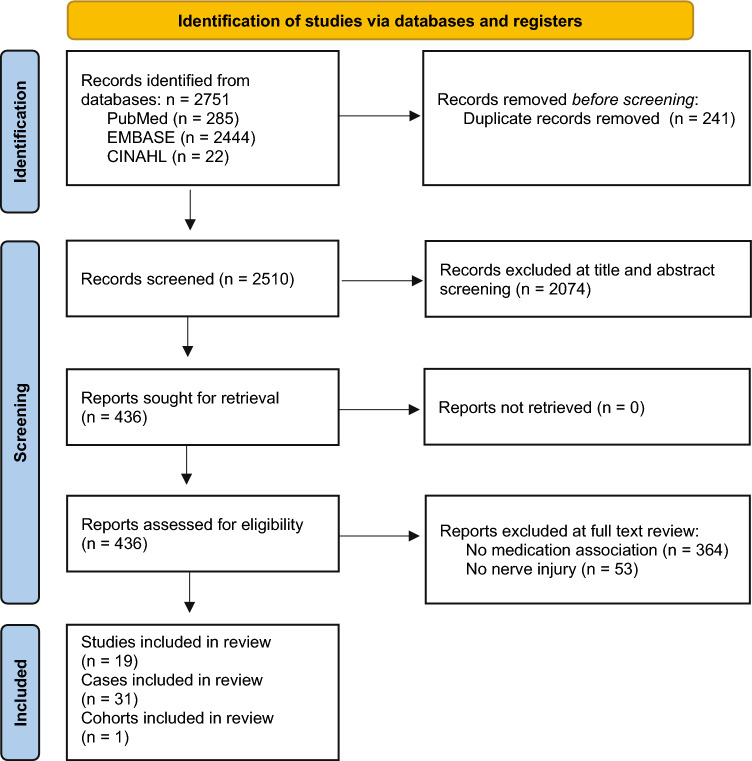
PRISMA flow diagram for peripheral nerve complications from medications utilized for weight loss. Source: Page MJ, et al. BMJ 2021;372:n71. https://doi.org/10.1136/bmj.n71. This work is licensed under CC BY 4.0. To view a copy of this license, visit https://creativecommons.org/licenses/by/4.0/

There were 2510 non-duplicated records found from database searching, and following screening processes 19 articles were identified (Fig. 1) [26, 32–49]. From these articles, there were 31 cases, primarily published from 2024 to 2026 with the exception of one case published in 2013. There was also a case control study including 103 individuals from the Mayo Clinic health care network. The cases were published in the United States of America (24), Australia (5), Hungary (1) and Italy (1). The mean JBI score of case reports was 6.0 (SD 1.4) with 29 studies considered moderate to high quality studies (5 out of 8 or greater). For the purpose of this review, the Mayo Clinic study was considered a cohort study with a JBI score of eight out of 11, as the control comparison only applied to a small proportion (five) of the cases and was not considered in this review. This study was reviewed separately.

### Case reports

There were 18 males and 13 females, with a median age of 55.0 years (IQR 17). Age was not reported in six cases. The individual’s weight prior to commencement of medication was reported in ten cases, with a median of 103.2 kg (IQR 14.9), and BMI reported in nine cases (median 32.1 kg/m^2^, IQR 6.4). The median pre-commencement HbA1c was 9.3% (IQR 6.1) reported from 15 cases. There were 17 cases that reported comorbid diabetes mellitus and four cases that reported insulin resistance. Other relevant comorbidities included: hyperlipidemia (5 cases), hypertension (5 cases), renal disease (2 cases), cardiovascular disease (2 cases). Two cases reported prior bariatric surgery (27 years and 14 years prior to commencing weight loss medication). There were three cases (1 acute, 2 subacute) with multiple nutritional deficiencies at the time of presentation (thiamine deficiency in all, one each with additional deficiencies of vitamin E, B1 or B6). There were two cases with diabetes mellitus who were also taking metformin; neither had documentation of B12 deficiency. No cases reported substance use. Data were limited for tobacco use (two cases were nonsmokers, one case had recently ceased), and for alcohol intake (denied in one case and rare in another).

The majority of cases (16 cases) reported use of GLP-1RAs (13 cases semaglutide, 2 cases dulaglutide, 1 case used an unspecified GLP-1RA), with 12 cases reporting dual GIP/GLP-1RA (tirzepatide), one case was taking empagliflozin and one case abused dinitrophenol. A further case was initially taking semaglutide and then switched to tirzepatide. No cases were found associated with phentermine, bupropion, topiramate or orlistat. Dose was not reported in most cases (22 cases). The median weight loss was 20.4 kg (IQR 15.2) reported from 27 cases, with one further case reporting a BMI reduction of 14.6 kg/m^2^ (65.6 to 51). Percentage weight loss was 21.8% (IQR 16.4) from 11 cases, and weight loss rate could be calculated in 19 cases, as a median of 3.7 kg per month (IQR 1.7). The median reduction in HbA1c was 4.7% (IQR 5.3) reported from 12 cases.

The median duration from commencement of medication to onset of symptoms was 150.0 days (IQR 135.0), with data available in 21 cases. Peripheral nerve complications had acute (10 cases), subacute (18 cases), chronic (1 case) and insidious (1 case) onsets. One case was asymptomatic and neuropathy was incidentally detected. In 26 cases there was documentation of appropriate screening of relevant differentials for peripheral nerve complications. Nerve conduction studies were performed in 27 cases, with two cases documenting patient refusal and in two cases nerve conduction was not performed. Of note, in one case the nerve conduction studies were considered normal but skin biopsy confirmed small fiber neuropathy [42]. In three cases, weight loss medications were already discontinued in three cases by onset of symptoms. In eight cases medication was discontinued once the complication was identified; in two cases medication was continued. Most often, this information was not reported (18 cases). See Table 1 for summary of included cases.

**Table 1:** Included cases

Author	Year	Age/Gender	Onset	Drug	Weight loss (kg)	Diabetes
Fibular neuropathy						
Pennington	2024	49F	Acute	GLP1R agonist	31.8	No
Tucker	2024	77M	Acute	Tirzepatide	27.7	IGT
Tucker	2024	56F	Acute	Semaglutide	18.6	Yes
Desai	2025	72F	Sub-acute	Semaglutide	N/A	No
Dhupati	2026	74M	Sub-acute	Semaglutide	13.6	No
Dhupati	2026	60F	Sub-acute	Tirzepatide	24.9	No
O’Gorman	2026	58F	Acute	Tirzepatide	30.0	Yes
O’Gorman	2026	85M	Sub-acute	Semaglutide	30.0	Yes
Tanyous	2026	55M	Acute	Tirzepatide	22.7	Yes
Tanyous	2026	78M	Incidental	Tirzepatide	13.6	IGT
Distal symmetric polyneuropathy						
Phillips	2013	19F	Insidious	Dinitrophenol	+ 4.1	No
Ali	2024	43M	Chronic	Semaglutide	N/A	Yes
Sahyouni	2024	50M	Sub-acute	Semaglutide	3.6	Yes
Shakeel	2025	59F	Sub-acute	Tirzepatide	63.5	Yes
Shakeel	2025	48F	Sub-acute	Tirzepatide	25.8	Yes
O’Gorman	2026	48M	Sub-acute	Semaglutide	5	No
O’Gorman	2026	45M	Acute	Semaglutide	8	No
Lumbosacral radiculoplexus neuropathy						
Donigan	2025	39M	Acute	Semaglutide	14.6 kg/m^2^ (BMI)	IGT
Chandrashekhar	2026	53–73^	Sub-acute	Dulaglutide	18.1	Yes
Chandrashekhar	2026	53–73^	Sub-acute	Semaglutide, Tirzepatide	18.1	Yes
Chandrashekhar	2026	53–73^	Sub-acute	Semaglutide	22.7	Yes
Chandrashekhar	2026	53–73^	Sub-acute	Dulaglutide	4.1	Yes
Chandrashekhar	2026	53–73^	Sub-acute	Tirzepatide	23.6	Yes
Chandrashekhar	2026	53–73^	Sub-acute	Tirzepatide	15.9	Yes
O’Gorman	2026	78M	Acute	Empagliflozin	7	Yes
Stabile	2026	62M	Acute	Tirzepatide	5.6%	No
Neuralgic amyotrophy						
Lesinszki	2026	39M	Sub-acute	Semaglutide	34	No
Wen	2026	60M	Sub-acute	Tirzepatide	20.4	IGT
*Others*						
Obialo	2025	43M	Acute	Tirzepatide	27.2	Yes
Zahir	2026	27F	Sub-acute	Semaglutide	45	Yes
Stolwyk	2025	52F	Sub-acute	Semaglutide	11.3	Yes

^50% of cohort were male

### Fibular neuropathy

There were 10 cases of fibular neuropathy (foot drop), with five cases occurring acutely, four cases subacutely and one found incidentally [26, 34, 35, 40,44, 46]. In seven cases neuropathy was unilateral. In three cases neuropathy was bilateral, with two cases reporting that this occurred consecutively and one case reporting concurrent occurrence. Painless onset of foot drop was reported in nine cases. Eight cases had fibular neuropathy with entrapment at the fibular neck detected by nerve conductionstudies, one case had non-localized fibular neuropathy and in the other case the individual refused nerve conduction studies. Three cases underwent nerve ultrasound, which was abnormal in two cases demonstrating increased vascularity or mild hypoechogenicity respectively of the fibular nerve at the fibular head. MRI was performed in two cases, showing a compressed fibular nerve in one case, and mild T2 hyperintensity of the fibular nerve in the other. Six cases were managed with conservative treatment, which generally included avoidance of leg crossing and an ankle–foot orthosis or splint. Surgery was performed on four cases. Three cases that were managed conservatively reported complete recovery on follow-up (range 1–4 months).

### Distal symmetric polyneuropathy

There were seven cases of distal symmetric polyneuropathy presenting with length-dependent paresthesia and numbness; two cases also reported pain [26, 32, 41, 42, 49]. Onset was acute (1 case), subacute (4 cases), chronic (1 case) and insidious (1 case). Two cases had simultaneous development of symptomatic upper limb entrapments with onset of distal symmetric polyneuropathy: one with bilateral ulnar neuropathy at the elbows and unilateral median neuropathy at the wrist, and the other case with bilateral median neuropathy at the wrists. Nerve conduction studies supported the diagnosis in five cases, were reported as normal in one case (with skin biopsy confirming loss of small nerve fibers) and were not performed in one case. Most (5 out of 7 cases) did not report treatment or follow-up. In the case reported by Phillips et al. nutritional support was provided and partial improvement noted after 24 months. Sahyouni et al. described a case where treatment of neuropathic pain improved outcome at 12 months.

### Lumbosacral radiculoplexus neuropathy

There were nine cases of lumbosacral radiculoplexus neuropathy, unilateral in three cases and bilateral in six cases [26, 33, 36, 50]. Eight cases presented classically with sudden onset pain followed by leg weakness, and one case had painless progressive weakness. Onset was acute in three cases and subacute in six cases. Nerve conduction and EMG were supportive in all cases where it was conducted, with one case refusing testing. MRI of the lumbosacral nerve roots and plexus was abnormal in four out of the five cases in which it was performed. In two cases glucocorticoid treatment (intravenous methylprednisolone in both cases, oral steroid taper in one case) improved pain and there was partial recovery of motor function. One case received nutritional supplementation, with partial improvement reported at nine months. Five cases received ‘supportive care’ (presumed rehabilitation), two noting partial improvement, with no improvement in one case, and two cases lost to follow-up. Treatment and outcome were not reported in one case.

### Neuralgic amyotrophy

There were two cases with neuralgic amyotrophy [37, 48]. Lesinszki et al. detail a 39-year-old male with 34 kg of weight loss over 8 months on semaglutide. He developed sudden onset of bilateral upper limb pain followed by asymmetric weakness of right triceps, bilateral finger extension and left grip 1 month later. He had previously had a similar episode of left-sided neuralgic amyotrophy. nerve conduction studies showed bilateral radial neuropathies. intravenous methylprednisolone and physical therapy were partially effective at reducing pain, but he remained significantly functionally impacted at one month follow-up. Wen et al. report a 60-year-old male with impaired glucose tolerance who lost 20.4 kg in 5 months while taking tirzepatide. He developed acute pain and paresthesia in the right shoulder and hand, followed by progressive weakness of shoulder and triceps muscles. EMG confirmed acute denervation in right C5 to C8 myotomes. Conservative management with analgesia and physical therapy was relatively ineffective, but cessation of tirzepatide at four weeks post-onset correlated with partial improvement in pain and weakness.

### Guillain–Barré syndrome

Obialo et al. provide a brief description (abstract only) of a 43-year-old male who presented with typical symptoms and signs of Guillain–Barré syndrome [38]. There were no identified triggers, but he had been receiving tirzepatide for 5 months, losing 27.2 kg in weight. MRI of brain and whole spine as well as cerebrospinal fluid analysis were consistent with the diagnosis, and response to treatment with intravenous immunoglobulin was prompt. He was discharged for rehabilitation and no further outcome was reported.

### Wernicke’s encephalopathy, antiphospholipid syndrome, severe diffuse sensorimotor axonal polyradiculopathy

Zahir et al. report an unusual case with multiple complications from weight loss [47]. A 37-year-old female had been treated for diabetes mellitus and obesity with semaglutide, losing 45 kg and improving HbA1c from 14 to 7% over 10 weeks. Weakness, numbness, and pain in the right lower limb were rapidly followed by similar symptoms in other limbs, blurred vision and confusion. Examination confirmed ocular dysmetria, limb ataxia, upper limb paresis, lower limb paraplegia and length-dependent sensory loss. She was found to have thiamine and B12 deficiencies and multiple small cerebral infarcts attributed to antiphospholipid syndrome (potentially unmasked by B12 deficiency). nerve conduction studies and EMG were consistent with severe sensorimotor axonal polyradiculoneuropathy. Sural nerve biopsy confirmed axonal neuropathy. Nutritional support did not result in improvement at 6 months.

### Amyotrophic lateral sclerosis (ALS)

Stolywyk et al. described a 52-year-old female with a diagnosis of ALS with initial revised ALS functional rating score (ALSFRS-r) of 45, with rate of progression calculated at 0.6 units per month [43]. She was commenced on semaglutide for type 2 diabetes nine months after ALS diagnosis. For the next 6 months there was minimal weight loss or change in clinical status. She then developed rapid weight loss of 11.3 kg within 3 months associated with marked progression in weakness. ALSFRS-r rate of progression peaked 5.6 units per month during this period. Despite cessation of semaglutide, she continued to deteriorate requiring gastrostomy and eventually tracheostomy.

### Mayo Clinic Cohort

This study identified 77 individuals with fibular neuropathy and 26 individuals with lumbosacral radiculoplexus neuropathy that occurred following exposure to a GLP-1RA or dual GIP/GLP-1RA medication [45]. This included: semaglutide (50 cases), dulaglutide (27 cases), liraglutide (16 cases), tirzepatide (12 cases) and exenatide (4 cases). The duration of medication exposure was a median of 15 months (range 1–112 months) for fibular neuropathy and a median of 6 months (range 3–35 months) for lumbosacral radiculoplexus neuropathy.

Of the individuals with fibular neuropathy, the median age was 59.4 years (25.6–88.1), 56 (72.7%) were female; 65 cases (84.4%) had diabetes and three cases had prediabetes. The median weight loss was 18.1 kg (range 8.2–45.4 kg), percentage weight loss 15.7% (range 3.0–37.0), weight loss rate 2.5 kg per month (no range reported) and median percentage reduction in HbA1c was 1.2 (range − 2.5–5.0).

The median age of those with lumbosacral radiculoplexus neuropathy was 62.6 years (34.1–62.6); 12 (44.4%) were female and 25 cases (92.6%) had diabetes. The median weight loss was 22.7 kg (range 9.2–76.7), percentage weight loss 13.9% (range 3.6–28.5), weight loss rate 3.7 kg per month (no range reported) and median percentage reduction in HbA1c was 2.4 (range 1.0–8.5).

There was no difference in total weight loss (*p* = 0.389) between those with fibular neuropathy and lumbosacral radiculoplexus neuropathy. However, percentage weight loss was greater in those with fibular neuropathy (*p* < 0.001) and reduction in HbA1c was greater than in those with lumbosacral radiculoplexus neuropathy (*p* < 0.001).

## Discussion

This is the first systematic review of peripheral nerve complications associated with weight loss medications reporting on 134 cases, primarily published over the last 3 years mirroring increased use of newer agents [13]. The complications observed were diverse, including entrapment neuropathy (especially fibular neuropathy), lumbosacral radiculoplexus neuropathy and distal symmetric polyneuropathy. Most occurred around the time of weight loss, within weeks of commencing medication. Compared with peripheral nerve complications seen with bariatric surgery, nutritional deficiencies were infrequently reported [15, 19]. However, the degree and acuity of weight loss, as well as the improvement of glycemic control described in the cases was significant. The mechanism of nerve injury associated with weight loss medication is likely to be multifactorial.

Obesity, especially centralized, is independently associated with increased risk of clinically significant peripheral nerve complications, especially peripheral neuropathy [51–54]. This is furthered with the addition of hyperglycemia, hyperinsulinemia, increases in triglycerides and systolic blood pressure [53, 55, 56]. Although the cases in this review did not have diagnosed neuronal injury before medication commencement, they may well have had an increased susceptibility to such, as substantial obesity-related neuronal damage can be present without clinical symptoms [54, 56, 57]. The dorsal root ganglia and sensory nerve endings in the skin are not covered by the protective blood nerve barrier and are susceptible to obesity-mediated inflammatory injury [52]. In addition, lipid metabolism and endoplasmic reticulum function of Schwann cells may be disrupted by free and long-chain fatty acids, which may also impact the peripheral nervous system microvasculature [52, 58].

The majority of cases included in this review had diabetes mellitus or insulin resistance. Weight loss may correct hyperglycemia and provoke diabetic lumbosacral radiculoplexus neuropathy or treatment-induced neuropathy in diabetes (TIND) [15, 45]. Mechanisms for these disorders remain unclear. Rapid induction of hypoglycemia can induce production of inflammatory cytokines, vascular injury and oxidative stress [59–62]. More speculatively, membrane sphingolipids have an emerging role in regulation of cell growth and survival, immune cell trafficking, and vascular and epithelial integrity [63], and sphingolipid metabolism is altered in insulin resistance and diabetes mellitus [64]. Nerve biopsy in cases of weight-loss associated distal symmetric polyneuropathy and lumbosacral radiculoplexus neuropathy has shown inflammatory infiltrates within the endoneurium, perineurium and epineurium of affected nerves, with prominent microvessel wall disruption and neovascularisation in some cases [15, 45, 65–67]. Neuronal tissue may be further laid susceptible with reductions in nerve growth factor, essential for the development and survival of neuronal cells, occurring with reductions in white adipose tissue [65, 68, 69].

Entrapment neuropathies arise in locations where the respective nerve is anatomically predisposed to compression or irritation [71]. They occur more frequently in those with diabetes, and in those with higher BMI [72–74]. There is a correlation between BMI and the amount of subcutaneous tissue overlying the fibular nerve at the fibular head, as well as the cross-sectional area of the nerve itself. However, rapid weight loss may precipitate entrapment at common sites [15]. Ultrasound of individuals who experienced weight loss-related fibular neuropathy showed loss of subcutaneous, perineural and intraneural fat, reducing physical protection for the nerve [75]. A similar mechanism might apply for ulnar neuropathy at the elbow, but it is unclear how these mechanisms might directly precipitate carpal tunnel syndrome.

Obese individuals have high rates of micronutrient deficiencies [76]. This may be furthered by medication induced caloric reduction and subsequent failure to meet nutrient dietary intake, altered gastric transit and unchanged poorly nutritive dietary intake patterns [77–79]. Deficiencies of calcium, iron, selenium, zinc, and vitamins D, B, E and A have been demonstrated in large cohorts and registries of individuals treated with GLP-1RAs, with other micronutrient deficiencies expected [79–82]. The peripheral nerve complications arising from deficiencies of vitamins including B1 (thiamine), B2 (riboflavin), B6 (pyridoxine), B9 (folate), B12 (cobalamin), E and of copper are well established [83–86] and are hypothesized to be the primary causative factor in sensory predominant distal symmetric polyneuropathy secondary to weight loss surgery [15]. While overt deficiency was infrequently noted in these cases, nutritional assessment was poorly documented. Nutritional deficiencies may play a larger role in cases with a prolonged onset.

With the exception of dinitrophenol [87], it is unlikely these medications had direct neurotoxic effects. It is thought GLP-1RAs imbue multiple neuroprotective effects including reduction of inflammation, protection against oxidative insult, enhancement of neuronal growth and reduction of apoptosis through cAMP-dependent signaling cascades [88–90]. GLP-1RAs may improve glycemic control and improve nerve structure and function in diabetic peripheral neuropathy [91, 92]. They may also reduce vascular risk factors for nerve injury [93]. Overall, weight loss, including when assisted by medications, results in stabilized existing neuronal injury and improved symptoms [94–98].

Given the diverse health impacts of obesity, it is important to emphasize there are substantial benefits to controlled weight loss. This study cannot assess prevalence, but given the total number of cases described, it is expected that peripheral nerve complications are an uncommon adverse effect of weight loss medications. However, the functional impact can be significant and recovery is not guaranteed. Therefore, with increasing use of weight loss medications, it is important to take a preventive approach to avoid peripheral nerve complications. This includes identifying individuals who may be at increased risk, particularly those with pre-diabetes, diabetes or the metabolic syndrome. The duration of diabetes is independently associated with developing peripheral neuropathy, so past medical history should be carefully collected [93, 99–101]. Ensuring adequate baseline nutritional status, and addressing existing micronutrient deficiencies and elevated triglycerides is important and has been effective in preventing the development of peripheral neuropathy after bariatric surgery [103]. It seems reasonable to limit the rate of reduction in HbA1c to ≤ 2% over 3 months [104] and aim for a steady rate of weight reduction, avoiding rapid loss (9 kg or more over a period of 2–3 months), and commencing regular weight-bearing exercise to maintain muscle mass [23, 24]. Avoidance of exercises that involve frequent squatting or stretching may be advised in the short term [105]. Regular clinical review, especially of nutritional status, may be needed for some time after weight loss, as the average time to neuropathy diagnosis was nine years in bariatric surgery cohorts [106].

There are specific limitations to be considered when interpreting the findings of this study’s results. Due to the nature of case series and individual case reports, the manifestations reported cannot be assumed to reflect the true incidence of each disorder. Case reports by definition highlight unexpected or unusual outcomes and thus are subject to significant publication bias and barriers to publication. Lack of awareness about potential nerve complications of weight loss may inhibit identification of such by providers; conversely, the increased interest and use of novel agents in this population may mean increased contact with medical care providers and subsequent identification of preexisting peripheral nerve disorders. There were limited data available in some cases, particularly for abstracts. This prevented application of formal adverse drug reaction scales [107].

## Conclusions

There is increasing evidence of peripheral nerve disorders occurring in association with medical treatment of weight loss, particularly with GLP-1RAs and GIP/GLP-1RAs. There are side effects with any treatment, and the potential peripheral nerve complications from weight loss must be balanced against the benefits of therapy. Determining and comparing the incidence rate of these complications for each of the GLP-1RAs and GIP/GLP-1RAs in prospective cohort studies will be important to understand the risk posed by these agents. Similarly, further work should aim to identify safe rates of weight loss and rates of normalization of hyperglycemia to minimize harm.

## Supplementary Information

Below is the link to the electronic supplementary material.

Supplementary file1 (DOCX 25 KB)

## Data Availability

The datasets used and/or analyzed during the current study are available from the corresponding author on reasonable request.
