# Supplementary material for: Peripheral nerve complications from medications utilised for weight loss: a systematic review
Source: J Neurol. 2026 Aug 3;273(8):503. doi: 10.1007/s00415-026-14048-w (PMC13433482; doi:10.1007/s00415-026-14048-w)
Supplement: Supplementary file 1 [file 415_2026_14048_MOESM1_ESM.docx]

**Supplementary Data**

Peripheral nerve complications from medications utilised for weight loss: A systematic review

Journal of Neurology

Authors: Cullen O’Gorman, Matilda O’Gorman, Nicola Warren. Corresponding author: Cullen O’Gorman

Department of Neurology, Mater Hospital, Raymond Terrace, South Brisbane, Queensland, Australia

Email: [c.ogorman@uq.edu.au](mailto:c.ogorman@uq.edu.au)

**Supplementary 1:**

**Search Terms, including MeSH Terms:**

Neuropathies, neuropathy, neuritis, neuralgic, neurological, plexopathies, plexopathy, lumbosacral, brachial, plexus, peroneal, fibular, slimmers, paralysis, palsy, palsies, palsied, peripheral, PN, DPN, diabetic, diabetes, TIND, “parsonage turner syndrome", amyotrophy, weakness, sensory.

GLP1, glucagon like peptide 1, dual, GIP, glucose dependent insulinotropic polypeptide, liraglutide, semaglutide, tirzepatide, dulaglutide, exenatide, lixisenatide, **retatrutide,** mazdutide, **cotadutide,** ehmt1 protein human, canagliflozin, dapagliflozin, empagliflozin, ertugliflozin, bexagliflozin, linagliptin, sotagliflozin, 2s 3r 4r 5s 6r 2 4 chloro 3 4 ethoxybenzyl phenyl 6 methylthio tetrahydro 2h pyran 3 4 5 triol, slc5a2 protein human, SGLT2, sodium-glucose, phentermine, dinitrophenol*, bitter orange, aurantium, synephrine, topiramate, bupropion, amfebutamone, naloxone, orlistat.

**Supplementary 2:**

**JBI Quality Assessment for Case Reports**

| **Author** | **Year** | **1** | **2** | **3** | **4** | **5** | **6** | **7** | **8** | **Total** |
| --- | --- | --- | --- | --- | --- | --- | --- | --- | --- | --- |
| Phillips | 2013 | 1 | 1 | 1 | 1 | 1 | 1 | 1 | 1 | 8 |
| Ali | 2024 | 1 | 1 | 1 | 0 | 1 | 0 | 0 | 1 | 5 |
| Sahyouni | 2024 | 1 | 1 | 1 | 1 | 1 | 1 | 1 | 1 | 8 |
| Tuker (1) | 2024 | 1 | 1 | 1 | 1 | 0 | 0 | 0 | 1 | 5 |
| Tuker (2) | 2024 | 1 | 1 | 1 | 1 | 1 | 1 | 0 | 1 | 7 |
| Pennington | 2024 | 1 | 1 | 0 | 1 | 1 | 1 | 1 | 1 | 7 |
| Desai | 2025 | 1 | 1 | 0 | 1 | 1 | 0 | 0 | 1 | 5 |
| Donigan | 2025 | 1 | 1 | 1 | 1 | 1 | 1 | 1 | 1 | 8 |
| Obialo | 2025 | 1 | 1 | 1 | 1 | 1 | 1 | 0 | 1 | 7 |
| Shakeel (1) | 2025 | 1 | 1 | 1 | 1 | 0 | 0 | 0 | 1 | 5 |
| Shakeel (2) | 2025 | 1 | 1 | 1 | 1 | 0 | 0 | 0 | 1 | 5 |
| Stolwyk | 2025 | 1 | 1 | 1 | 1 | 1 | 1 | 1 | 1 | 8 |
| Chandrashekhar (1) | 2026 | 0 | 0 | 1 | 1 | 1 | 1 | 1 | 1 | 6 |
| Chandrashekhar (2) | 2026 | 0 | 0 | 1 | 1 | 0 | 1 | 1 | 1 | 5 |
| Chandrashekhar (3) | 2026 | 0 | 0 | 1 | 1 | 0 | 1 | 1 | 1 | 5 |
| Chandrashekhar (4) | 2026 | 0 | 0 | 1 | 1 | 0 | 1 | 1 | 1 | 5 |
| Chandrashekhar (5) | 2026 | 0 | 0 | 1 | 1 | 0 | 0 | 0 | 1 | 3 |
| Chandrashekhar (6) | 2026 | 0 | 0 | 1 | 1 | 0 | 0 | 0 | 1 | 3 |
| Dhupati (1) | 2026 | 1 | 1 | 1 | 1 | 1 | 0 | 0 | 1 | 6 |
| Dhupati (2) | 2026 | 1 | 1 | 1 | 1 | 1 | 0 | 0 | 1 | 6 |
| Lesinski | 2026 | 1 | 1 | 1 | 1 | 1 | 1 | 0 | 1 | 7 |
| O'Gorman (1) | 2026 | 1 | 1 | 1 | 1 | 0 | 0 | 0 | 1 | 5 |
| O'Gorman (2) | 2026 | 1 | 1 | 1 | 1 | 0 | 0 | 0 | 1 | 5 |
| O'Gorman (3) | 2026 | 1 | 1 | 1 | 1 | 0 | 0 | 0 | 1 | 5 |
| O'Gorman (4) | 2026 | 1 | 1 | 1 | 1 | 0 | 0 | 0 | 1 | 5 |
| O'Gorman (5) | 2026 | 1 | 1 | 1 | 1 | 0 | 0 | 0 | 1 | 5 |
| Stabile | 2026 | 1 | 0 | 1 | 1 | 1 | 1 | 0 | 1 | 6 |
| Tanyous (1) | 2026 | 1 | 1 | 1 | 1 | 1 | 1 | 1 | 1 | 8 |
| Tanyous (2) | 2026 | 1 | 1 | 1 | 0 | 1 | 1 | 1 | 1 | 7 |
| Wen | 2026 | 1 | 1 | 1 | 1 | 1 | 1 | 1 | 1 | 8 |
| Zahir | 2026 | 1 | 1 | 1 | 1 | 1 | 1 | 1 | 1 | 8 |

Adapted criteria

1: Were the patient’s demographic characteristics clearly described?

2: Was the past history clearly reported?

3: Was the current clinical condition/presentation clearly reported?

4: Were diagnostic tests or assessment methods reported?

5: Was an intervention or treatment reported?

6: Was a post-intervention or outcome of the presentation reported?

7: Was a follow up period reported on?

8: Does the case report contain a takeaway lesson?

**JBI Quality Assessment for Cohort Studies**

| Author | Year | 1 | 2 | 3 | 4 | 5 | 6 | 7 | 8 | 9 | 10 | 11 | Total |
| --- | --- | --- | --- | --- | --- | --- | --- | --- | --- | --- | --- | --- | --- |
| Triplett | 2025 | 1 | 1 | 1 | 1 | 0 | 1 | 1 | 1 | 0 | 0 | 1 | 8 |

Adapted criteria

1: Were the two groups similar and recruited from the same population?

2: Were the exposures measured similarly to assign people to both exposed and unexposed groups?

3: Was the exposure measured in a valid and reliable way?

4: Were confounding factors identified?

5: Were strategies to deal with confounding factors stated?

6: Were the groups/participants free of the outcome at the start of the study?

7: Were the outcomes measured in a valid and reliable way?

8: Was the follow up time reported and sufficient to be long enough for outcomes to occur?

9: Was follow up complete, and if not, were the reasons to loss to follow up described and explored?

10: Were strategies to address incomplete follow up utilized?

11: Was appropriate statistical analysis used?
